# Supplementary material for: Differences in Sodium Channel Densities in the Apical Dendrites of Pyramidal Cells of the Electrosensory Lateral Line Lobe
Source: Front Neural Circuits. 2019 Jun 4;13:41. doi: 10.3389/fncir.2019.00041 (PMC6558084; doi:10.3389/fncir.2019.00041)
Supplement: Supplementary file 1 [file Data_Sheet_1.pdf]

**Supplemental Material**

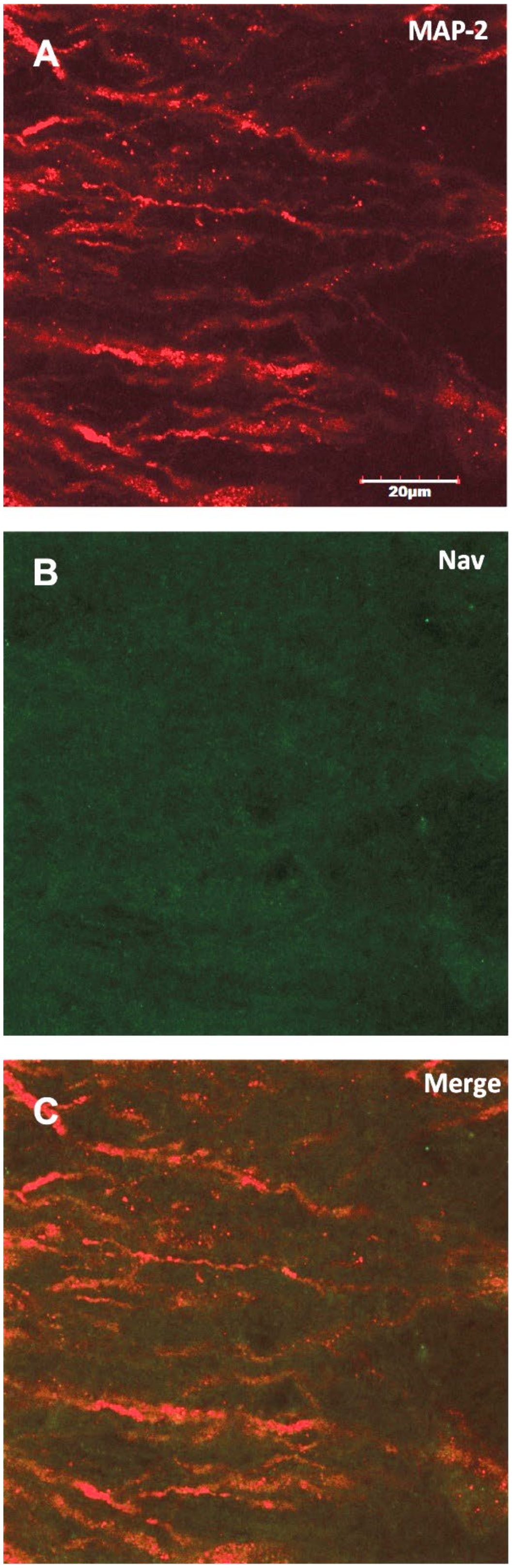


**Supplemental Figure 1. Absorption control:** Confocal scan showing apical dendrites stained by Anti-MAP2 marker **(A)** in the ELL-LS segment. Anti-pan Nav antibody was incubated with immunogen (peptide) in a 1:10 ratio overnight on a nutator at 4 degrees. Slide mounted ELL sections were incubated with pre-adsorbed Nav antibody along with Anti-MAP 2 primary antibody which was followed by incubation with their individual secondary antibodies. No Nav expression was observed **(B,C)** demonstrating the specificity of Nav antibody used. Scalebar – 20 µm ( 60X objective, **2X** digital Zoom). All scanning parameters and other steps of the protocol where identical to the ones used in the results section. Note that brightness of the image was increased to see the autofluorescence otherwise the image would have been completely black.

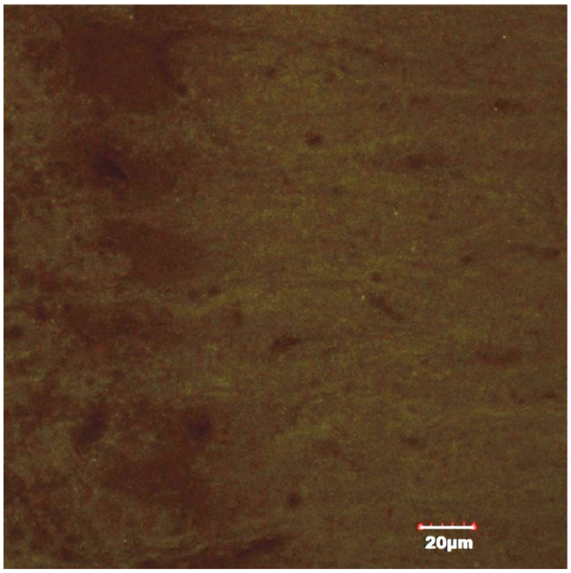


**Supplemental Figure 2. No primary antibody control :** Anti-pan Nav and Anti MAP-II antibody used for labelling Nav and microtubules respectively were omitted from the IHC protocol as a control for non-specific binding of the secondary antibodies. Scalebar – 20 µm ( 60X objective, **1X** digital Zoom). Note that brightness of the image was increased to see the autofluorescence otherwise the image would have been completely black.

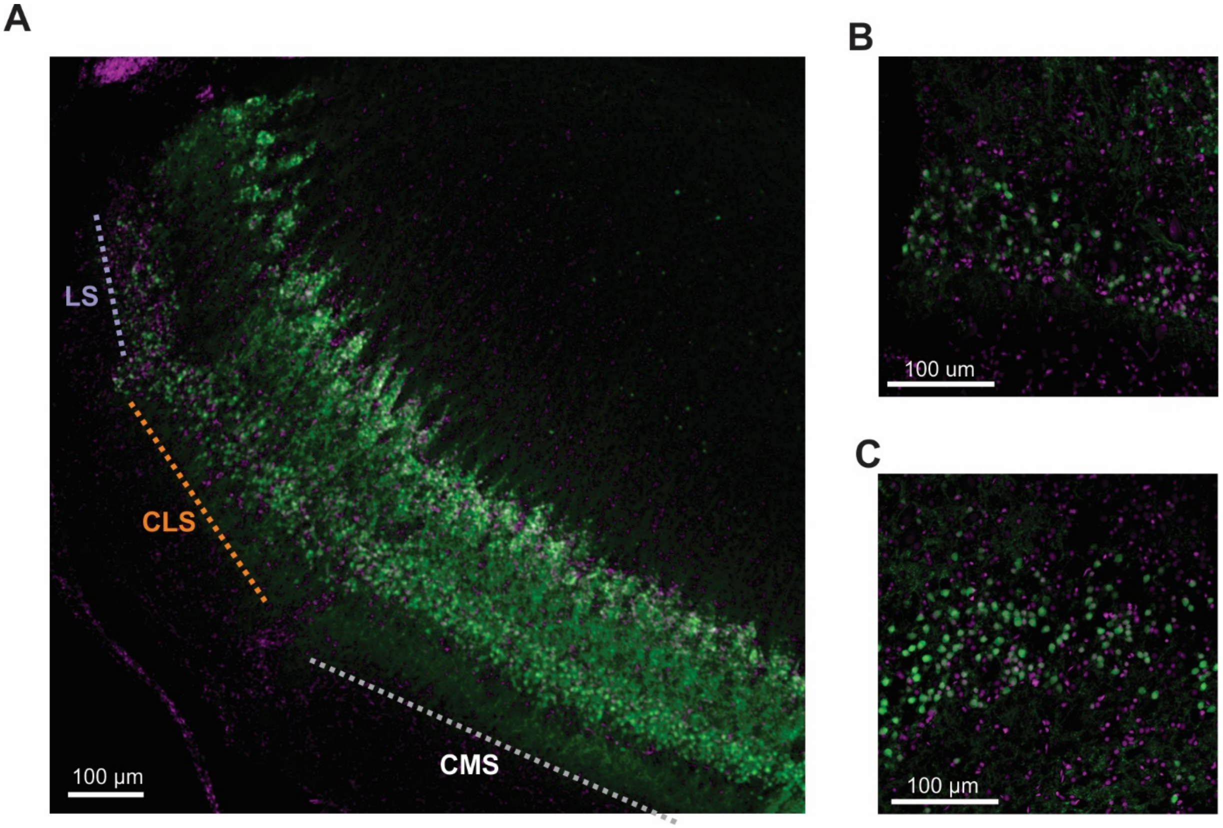


**Supplemental Figure 3: Gradient across ELL segments in number of GABA-labeled cell bodies.**

**A.** As described in the discussion, GABA may influence spiking dynamics. Preliminary data shown here (N=1 animal, n=4 sections sampled) indicate that the distribution of GABAergic interneurons vary across ELL segments. 10x magnification of ELL stained with Sigma Aldrich Anti-GABA produced in rabbit antibody (Sigma Aldrich # A2052) with Goat anti rabbit Alexa 488 secondary (Life Technologies, #A-11008; green) and a nuclear marker (Syto 59, Lifetechnologies #s11341; magenta) show a gradient of GABA distribution across segments. **B.** 40x magnification of LS and CLS (**C**). Only LS and CLS data have been quantified. We counted the number of GABA-labeled cell bodies in the granular cell layers in 100µm x 100µm area. Overall differences in mean number of interneurons were not found (LS=161.77 CLS=193.83, p=.52) but the variation in interneurons found was much higher for CLS, suggesting that the upper limit of interneurons found in this section is higher (standard deviations: LS=47.2726, CLS=137.5361)
